# Obstetric Anal Sphincter Injury Care Bundle: A Quality Improvement Initiative

**DOI:** 10.1007/s00192-024-05885-2

**Published:** 2024-09-27

**Authors:** T. Clark Powell, Tanya P. Hoke, Kyle P. Norris, Margaret R. Page, Allison Todd, David T. Redden, Cynthia G. Brumfield, J. Michael Straughn, Holly E. Richter

**Affiliations:** 1 Division of Urogynecology and Reconstructive Pelvic Surgery, Department of Obstetrics & Gynecology, University of Alabama at Birmingham, 1700 6Th Ave S, 176F Suite 10382, Birmingham, AL 35233 USA; 2 Department of Obstetrics & Gynecology, University of Alabama at Birmingham, Birmingham, AL USA; 3 Center for Clinical and Translational Science, University of Alabama at Birmingham, Birmingham, AL USA; 4 Division of Maternal-Fetal Medicine, Department of Obstetrics & Gynecology, University of Alabama at Birmingham, Birmingham, AL USA; 5 Division of Gynecologic Oncology, Department of Obstetrics & Gynecology, University of Alabama at Birmingham, Birmingham, AL USA

**Keywords:** Clinical care bundle, Obstetric anal sphincter injury, Quality improvement

## Abstract

**Introduction and Hypothesis:**

The objective was to implement an evidence-based peri-partum care bundle for women sustaining obstetric anal sphincter injuries and to evaluate compliance with recommendations for antibiotics use, repair in the operating room, and follow-up before and after implementation.

**Methods:**

This project was reviewed by the Institutional Review Board and determined to be exempt. A clinical care bundle containing education and standardized orders in the electronic medical record was implemented. Characteristics of pre- (October 2017 to September 2019) and post-intervention (October 2019 to August 2021) cohorts were compared and compliance with recommendations for antibiotics use, surgical repair location, and follow-up were evaluated. Chi-squared, Fisher’s exact, ANOVA F, and Kruskal–Wallis tests were performed, as indicated. Significance level was *p* < 0.05.

**Results:**

A total of 185 cases were identified. Seventy-five percent of women were nulliparous. Mean gestational age was 39 weeks. Pre- and post-intervention groups did not differ in age, BMI, race, parity, gestational age, comorbidities, birthweight, or delivery type. Ninety-eight cases were identified pre-implementation. Eighty-six (88%) had third-degree lacerations. Post-implementation, 87 cases were identified. Seventy (80%) had third-degree lacerations (*p* = 0.17). Recommended antibiotic-type use improved from 35% pre-implementation to 93% post-implementation (*p* < 0.001). Repair in the operating room was similar pre-implementation and post-implementation (16.0% vs 12.6%, *p* = 0.48). Post-partum follow-up within 2 weeks improved from 16.3% pre-implementation to 52.8% post-implementation and mean time to follow-up was shorter post-implementation than pre-implementation (18 vs 33 days; both *p* < 0.001).

**Conclusions:**

Implementation of an evidence-based peri-partum care bundle resulted in standardization of care in accordance with established recommendations. Compliance with recommendations for surgical repair in the operating room remained unchanged.

**Supplementary Information:**

The online version contains supplementary material available at 10.1007/s00192-024-05885-2

## Introduction

Obstetric lacerations occur in 75% of vaginal deliveries and damage to the anal sphincter complex occurs in approximately 6–7% of these [1]. Obstetric anal sphincter injuries (OASIS) may increase the risk of OASIS in subsequent deliveries, result in loss of bowel control, and negatively affect quality of life [2, 3]. Furthermore, they have an effect on post-partum recovery, mental health, and choices about future deliveries [4]. OASIS are tracked as a National Quality Indicator for obstetric care (National Quality Forum #0748) [5]. Risk factors for OASIS (operative delivery, episiotomy, increased fetal birthweight, labor induction/augmentation, persistent occiput posterior fetal position, and primiparity) [6] are not universally modifiable for prevention. However, there are opportunities for optimizing OASIS recognition, repair and post-partum care to achieve the best possible outcomes. Unfortunately, there is a lack of guidelines or complete consensus on which are the most important elements of peri-partum OASIS care [7]. Wound infection or breakdown is common following OASIS and risk is mitigated by administering appropriate preoperative antibiotics (second-generation cephalosporin) [8, 9]. Further, these complications most often present within 2 weeks after delivery [8]. Many guidelines suggest surgical repair in an operating room given the importance of adequate lighting, anesthesia, and appropriate instruments in achieving excellent anatomical and functional repair [7]. Although long-term outcomes with regard to continence, prolapse, and pelvic floor function are difficult to study and quantify after OASIS, it is clear that preventing infection, breakdown, and reoperation may contribute to mitigating these undesirable outcomes.

This evidence-based quality improvement (QI) project resulted from a recognition that OASIS management was not standardized and that, as a result, not all patients were receiving optimal care. QI is an ongoing endeavor whereby opportunities to enhance clinical care and outcomes are identified by either an anecdotal or iterative process followed by an intervention whose outcomes are then evaluated. We sought to integrate input from clinical leaders and stakeholders with available evidence-based recommendations to affect positive change and achieve appropriate standardization in our clinical management [10, 11]. Care bundles are used widely in QI projects and have been shown to outperform individual interventions [12, 13]. We sought to optimize peri-partum management of OASIS in an evidence-based fashion with particular attention to antibiotic administration at the time of repair, repair in an operating room rather than in a labor suite, and close-interval follow-up within 2 weeks. The primary aim of this study was to evaluate the effectiveness of a departmental QI initiative aimed at increasing adherence to best practices in these domains and to compare outcomes with pre-QI management. We hypothesized that an evidence-based peri-partum clinical care bundle would improve standardization of care and compliance with management recommendations.

## Materials and Methods

This project was evaluated by the Institutional Review Board and determined to be exempt. Following a review of the literature (Appendix 1) regarding prevention, surgical repair, and post-partum management of OASIS, a multidisciplinary team of obstetrician/gynecologists (including maternal–fetal medicine, gynecological oncology, and urogynecology subspecialists), nursing leaders, and quality officers developed a peri-partum QI care bundle for women who sustain OASIS during vaginal delivery. Women who sustained a third- or fourth-degree laceration during childbirth from January 2017 to September 2019 constituted the pre-QI care plan cohort. Baseline demographic as well as intra-partum and post-partum variables including return to the OR, antibiotics utilized, wound issues, and any other patient complaints were identified and collected into the OASIS database. In October 2019, after a review of the literature, an evidence-based curriculum was introduced in the form of didactic instruction and written educational material for nurses, attending physicians, fellows, and residents regarding optimal intra-partum care, recognition, diagnosis, surgical repair, and post-partum care of OASIS (Appendix 1). Educational meetings were held for all stakeholders during already established educational conference time. Experienced providers taught surgical repair simulation sessions using a previously described beef tongue model [14]. These didactic sessions were then repeated twice yearly. A standardized surgical and post-partum care bundle was also implemented via an order set within the electronic medical record (Appendix 2). Following the initial pre-/post-implementation period, a quarterly quality monitoring system was created that reviewed all OASIS cases to ensure that the appropriate order set was used and follow-up was scheduled. Women were scheduled in a post-partum OASIS follow-up clinic staffed by fellowship-trained urogynecologists 2 weeks after delivery in order to identify any developing wound complications, provide additional counseling, and collect patient-reported outcomes (these patient-reported outcomes will be a part of another report).

The post-QI care plan implementation cohort consisted of women sustaining OASIS from October 2019 through August 2021. Demographic, intra-partum, and post-partum outcome data were prospectively collected to compare adherence to evidence-based practices for antibiotics use (second-generation cephalosporin), surgical repair in the OR, and close-interval follow-up (within 2 weeks) before and after QI bundle implementation. Although this study was not powered to compare clinical outcomes between groups, any need for intervention such as treatment with antibiotics or wound revision was tracked. Chi-squared or Fisher’s exact tests for categorical data, and ANOVA F or Kruskal–Wallis tests for continuous data, were performed as appropriate. Significance level was *p* < 0.05.

## Results

A total of 185 cases of OASIS were identified over the entire QI initiative timeline. Overall demographic and clinical characteristics of the patients are noted in Table 1. Mean age was 27.6 ± 6 years and BMI was 27.6 ± 6 kg/m^2^. Seventy-five percent of women were nulliparous and mean gestational age was 39 weeks. The majority of women self-identified as Black (54, 29%) or white (80, 43%). One hundred and seventeen out of 185 deliveries (63%) were spontaneous. Operative deliveries were performed in 68 (58%) of the total cases and these were predominantly performed using forceps (*n* = 58). Vacuum delivery was performed in 12 cases (6%). Mean birthweight was 3,444.8 ± 493 g and average estimated blood loss (EBL) from delivery was 497 ± 271 ml (*p* = 0.77 and 0.57 respectively).

**Table 1:** Patient clinical and demographic characteristics

Characteristics, *n* (%)	Total (185)	Pre-QI (98)	Post-QI (87)	p*, **
Age, mean (SD)	27.59 ± 5.99	27.68 ± 6.05	27.49 ± 5.96	0.83
BMI, mean (SD)	26.56 ± 5.84	27.10 ± 6.00	25.71 ± 5.57	0.13
Race, %				
Black or African American	54 (29.19)	25 (25.51)	29 (33.33)	0.44
White	80 (43.24)	46 (46.94)	34 (39.08)	
Other	51 (27.57)	27 (27.55)	24 (27.59)	
Diabetes, %	11 (5.95)	6 (6.12)	5 (5.75)	0.91
Hypertension, %	7 (3.18)	3 (3.06)	4 (4.60)	0.56
Current smoker, %	5 (2.70)	3 (3.06)	2 (2.30)	0.75
Parity				
0	139 (75.14)	70 (71.43)	69 (79.31)	0.34
1	31 (16.76)	19 (19.39)	12 (13.79)	
2	14 (7.57)	9 (9.18)	5 (5.75)	
3	1 (0.54)	0 (0.00)	1 (1.15)	
Gestational age, weeks	38.99 ± 1.23	39.11 ± 1.44	38.85 ± 0.93	0.14
Induction of labor	119 (64.32)	57 (58.16)	62 (71.26)	0.06
VBAC	11 (5.95)	8 (8.16)	3 (3.45)	0.18
Duration of second stage, min	94.32 ± 81.26	99.29 ± 85.17	88.64 ± 76.75	0.38
Delivery type				
Spontaneous	117 (63.2)	60 (61.2)	57 (65.5)	0.09
Forceps	56 (30.3)	28 (28.6)	28 (32.2)	
Vacuum	12 (6.5)	10 (10.2)	2 (2.3)	
Episiotomy				
None	178 (96.74)	96 (97.96)	82 (95.35)	0.51
Mediolateral	1 (0.54)	0 (0.00)	1 (1.16)	
Midline	5 (2.72)	2 (2.04)	3 (3.49)	
Laceration type				
Third degree	156 (84.52)	86 (87.76)	70 (80.46)	0.17
Fourth degree	29 (15.68)	12 (12.24)	17 (19.54)	
Birthweight, g	3,444.8 ± 493.0	3,435.8 ± 522.9	3,457.9 ± 449.3	0.77
Estimated blood loss, ml	496.6 ± 270.7	507.2 ± 307.3	484.7 ± 224.1	0.57

*VBAC* vaginal birth after cesarean, *QI* quality initiative

*Chi-squared or Fisher’s exact test for count data unless otherwise noted, ANOVA F test for mean (SD) data, Kruskal–Wallis test for median data

**p value reflects comparisons of the pre- and post-QI groups

Pre- and post-intervention groups did not differ with regard to mean age, BMI, race, parity, gestational age, or comorbidities (all *p* > 0.05, Table 1). Ninety-eight OASIS cases occurred prior to care bundle implementation. Of these, 86 (88%) were third-degree lacerations. After care bundle implementation, 87 OASIS cases were identified. Of these, 70 (80%) were third-degree lacerations (*p* = 0.17). There were similar rates of labor induction and vaginal birth after cesarean (VBAC). There was a trend toward significance (*p* = 0.06) for increased induction of labor in the post-implementation group. Additionally, birthweight, duration of second stage of labor, delivery type (spontaneous, forceps, vacuum), and EBL did not differ significantly pre- versus post-intervention (Table 1).

Recommended standard-of-care antibiotic use improved from 35% (31 out of 98) pre-bundle to 93% (83 out of 87) post-bundle (*p* < 0.001, Table 2). Repair in the operating room was similar pre- and post-intervention [15] (16.0%, 16 out of 98 vs 12.6%, 11 out of 87; *p* = 0.48). Follow-up within 2 weeks improved from 16.3% pre-intervention to 52.8% post intervention (*p* < 0.001) and mean time to follow-up was shorter pre-intervention than post-intervention (33.1 vs 18.3 days; both *p* < 0.001; Table 2). Need for post-partum intervention was low pre- and post-care bundle implementation.

**Table 2:** Outcomes after implementation of quality initiative (QI) bundle

Characteristics, *n* (%)	Total (185)	Pre-QI (98)	Post-QI (87)	p*
Antibiotics at time of repair	114 (61.6)	31 (31.6)	83 (95.4)	< 0.001
Cefotetan	88 (77.19)	11 (35.48)	77 (92.77)	< 0.001
Ceftriaxone	22 (19.30)	16 (51.61)	6 (7.23)	
Cefazolin	4 (3.51)	4 (12.90)	0 (0.00)	
Repair in operating room	27 (14.59)	16 (16.33)	11 (12.64)	0.48
Follow-up within 2 weeks, %	62 (33.5)	16 (16.3)	46 (52.8)	< 0.001
Time to follow-up, days	26.19 ± 5.99	33.08 ± 15.37	18.26 ± 14.11	< 0.001
Post-partum intervention				
None	177 (95.69)	95 (96.94)	82 (94.25)	0.39
Oral antibiotics	5 (2.70)	1 (1.02)	4 (4.60)	
Surgical revision	3 (1.62)	2 (2.04)	1 (1.15)	

*Chi-squared or Fisher’s exact test for count data unless otherwise noted, ANOVA F test for mean (SD) data, Kruskal–Wallis test for median data

*QI* quality initiative

## Discussion

This QI initiative sought to understand current care and potentially improve care for women sustaining OASIS by standardizing peri-partum management in accordance with evidence-based practice recommendations. This was attempted by creating a clinical care bundle that included department-wide education, simulation, and an order set built into the electronic medical record that facilitated ordering of recommended preoperative antibiotics as well as outpatient follow-up clinic scheduling. Following the OASIS care bundle implementation, significant improvements were seen in compliance with use of evidence-based recommended antibiotics and timing of post-partum follow-up. However, rates of repair in an operating room rather than in the delivery suite remained low and unchanged.

Overall, the pre-/post-OASIS care bundle implementation groups were well matched, decreasing the risk of overt confounding. However, there was a trend toward significance for increased induction of labor in the post-implementation group, potentially reflecting the ARRIVE (A Randomized Trial of Induction Versus Expectant Management) trial data, which provided evidence for improved maternal and fetal outcomes with induction of labor at 39 weeks [15].

Appropriate antibiotics administration has been shown to decrease rates of post-partum wound complications [8]. This was demonstrated in a randomized controlled trial by Duggal et al., during which a single dose of a second-generation cephalosporin yielded decreased wound infection at 2 weeks post-partum compared with placebo [9]. Observational studies have also provided evidence for reduced risk of perineal wound complications, including infection and breakdown, when antibiotics are given prior to or at the time of repair [8, 16]. Both the American College of Obstetricians and Gynecologists and the Royal College of Obstetricians and Gynaecologists recommend broad-spectrum antibiotics administration for OASIS repair [17, 18]. However, at our institution, rates of appropriate antibiotics administration had been low. Other investigations have highlighted barriers to guideline adherence that are as simple as awareness or remembering to place an order [19]. Therefore, we provided training, an online educational handout with references, and built appropriate antibiotics orders into the OASIS care bundle within the electronic medical record. As a result, use of the recommended antibiotics improved from 35 to 93%. We expect this change to be sustained as usage of order sets in the electronic medical record is built into the workflow on labor and delivery.

Location of OASIS repair is not well studied but using an operating room is recommended widely in published national guidelines [7]. Although not included in the ACOG recommendations, leaders in our institution including stakeholders from Nursing, Institutional Quality, and Obstetrics and Gynecology (Generalists, Maternal–Fetal Medicine, Urogynecology, and Gynecologic Oncology) agree that repair in the operating room rather than a labor suite would improve exposure, lighting, analgesia, assistance, and access to appropriate instruments. Therefore, the recommendation for all repairs to be performed in an operating room was included in both the didactic and written portions of the QI bundle. In spite of this, there was no significant change in rates of repair in the operating room 16% pre- and 13% post-intervention. This likely represents an as-yet unidentified barrier to moving a patient from the labor suite to the operating room and may be multifactorial, potentially including the desire to leave operating rooms available for emergency cesarean deliveries or the perception that the time and personnel required to move the patient outweighs the benefit of operating room resources. Some providers have elected to use mobile lighting or additional sterile instrument trays from the operating room in the labor suite rather than transporting the patient. As such, further investigation is warranted to determine if the benefits of moving to an operating room outweigh the perceived or unintended consequences (delay in repair, increased blood loss, patient discomfort). This decision may be institution specific or driven by other concomitant events occurring during labor and delivery.

Close-interval follow-up after OASIS has been identified as a means of improving outcomes by allowing for mitigation and management of post-partum perineal complications including infection and breakdown, which have been as high as 20% in some investigations and most often present within 2 weeks of delivery [8]. Additionally, this serves as an opportunity for evaluation and education regarding continued bowel management to optimize the patient experience and outcomes. At our institution, prior to the QI initiative, post-partum follow-up timing has been left to individual providers or based on institutional guidelines for the management of comorbidities (hypertension, diabetes, etc.). To facilitate post-partum follow-up for women with OASIS, the peri-partum quality bundle included awareness education as well as automatic scheduling within 2 weeks post-partum in a clinic staffed by urogynecology providers. Rate of follow-up within 2 weeks improved from 16% pre- to 53% post-intervention. This scheduling is dependent on patient-level variables that are not accounted for (patient preference, socioeconomic, transportation, and demographic factors); therefore, we also assessed average time to post-partum follow-up, which was significantly shorter post-intervention (33 vs 18 days). It is important to note that chart review confirmed that as a result of this intervention, all individuals who sustained OASIS were contacted and offered follow-up within the appropriate time frame. As with antibiotics administration, this improvement was achieved by including the scheduling within a standardized order set. Therefore, we expect it to be sustained over time.

This QI initiative is descriptive and its purpose was not to detect differences between groups with regard to infection, wound breakdown, or other complications. However, the improved rate of administration of perioperative antibiotics and close-interval follow-up will hopefully serve to mitigate these complications in a large obstetric population, as has been seen in other studies. Future research initiatives are addressing this outcome.

Further investigation is warranted to continue to elucidate the details of peri-partum care that can reduce the incidence of OASIS and further avoid its short- and long-term consequences. Additional study and interval collection of quality data may demonstrate other factors that can be optimized to improve outcomes and that could then be added to the care bundle.

Strengths of this project include a large number of women undergoing delivery at a large institution with the ability to implement changes via the electronic medical record—an approach that could be replicated elsewhere.

There are limitations to the conclusions that can be drawn from this QI project. First, QI can be dependent on the setting in which change is attempted, which limits external validity. However, bundled care and facilitating best-practices by building them into the electronic medical record has been shown elsewhere to provide effective, durable change [12]. Although data were collected on post-partum outcomes, including infection or surgical revision, the purpose of this investigation was not to compare these outcomes between groups. Finally, as a report summarizing this quality improvement initiative with its focus on studying change in peri-partum care, our goal was not to report longer-term clinical, including pelvic floor, outcomes. However, patients are currently being followed-up 6 weeks, 3, 6, and 12 months post-delivery where validated symptom distress and impact questionnaires are being collected.

Implementation of the OASIS care bundle significantly improved antibiotics administration and appropriate post-partum follow-up timing at a quaternary care institution. However, it did not affect compliance with recommendations for OASIS repair to be performed in an operating room rather than in the labor suite. This study highlights the value of care bundles and the power of the electronic medical record in reducing barriers to compliance with evidence-based practice. Further investigation is warranted to determine if these interventions result in improved outcomes and to identify other interventions that could reduce actual OASIS rates and continue to optimize outcomes.

## Supplementary Information

Below is the link to the electronic supplementary material.

Supplementary file1 (PDF 515 KB)

Supplementary file2 (PDF 646 KB)
