# Supplementary material for: Obstetric Anal Sphincter Injury Care Bundle: A Quality Improvement Initiative
Source: Int Urogynecol J. 2024 Sep 27;35(11):2125–30. doi: 10.1007/s00192-024-05885-2 (PMC11638415; doi:10.1007/s00192-024-05885-2)
Supplement: Supplementary file 1 — Supplementary file1 (PDF 515 KB) [file 192_2024_5885_MOESM1_ESM.pdf]

Appendix 1.
2-23-24

Obstetric Anal Sphincter Injuries (OASIS) Management Recommendations

Introduction

Obstetric Anal Sphincter Injuries (OASIS) occur overall in approximately 6-7% of vaginal deliveries[1] and may result in loss of bowel control[2-3]. As of 2003, OASIS was considered a National Quality Indicator (National Quality Forum #0748)[4]. It is important for women at risk (vaginal delivery, operative vaginal delivery, episiotomy, increased fetal birth weight, labor induction/augmentation, persistent OP fetal position and primiparity)[5] to be identified to try to optimize prevention strategies. For women who sustain an injury, it is vital that they are managed in an evidence-based manner to minimize morbidity. An OASIS classification system is noted below. We present the evidence supporting active management with respect to prevention and management of these obstetric injuries.

Box 1. Classification of Perineal Lacerations

First degree: Injury to Perineal skin only.

Second degree: Injury to perineum involving perineal muscles but not involving anal sphincter.

Third degree: Injury to perineum involving anal sphincter complex.
3a: Less than 50% of external anal sphincter thickness torn.
3b: More than 50% external anal sphincter thickness torn.
3c. Both external anal sphincter and internal sphincter torn.

Fourth degree: Injury to perineum involving anal sphincter complex (external anal sphincter and internal anal sphincter) and anal epithelium.

Modified from American College of Obstetricians and Gynecologists. Obstetric data definitions (version 1.0). Washington, DC: American College of Obstetricians and Gynecologists; 2014. Available at https://www.acog.org/-/media/Departments/Patient-Safety-and-Quality-Improvement/2014reVITALizeObstetricDataDefinitionsV10.pdf. Retrieved April 29, 2016.

Intrapartum Management: Potential Prevention

Perineal Massage

Perineal massage (antepartum or during the second stage of labor) is intended to decrease perineal muscular resistance and reduce the likelihood of laceration at

delivery. A meta-analysis of data from two studies (2,147 women) found that perineal massage during the second stage of labor reduced third-degree and fourth-degree tears when compared with “hands off” the perineum (RR, 0.52; 95% CI, 0.29–0.94), but was not associated with significant changes in the rate of birth with an intact perineum.[6]

Warm compress

A meta-analysis of two studies (1,525 women) that randomized participants to warm compresses on the perineum during the second stage of labor versus no warm compresses found that compress use significantly reduced third-degree and fourth-degree lacerations (RR, 0.48; 95% CI, 0.28–0.84). However warm compresses did not increase the rate of a woman having an intact perineum after delivery (RR, 1.05, 95% CI, 0.86–1.26).[6]

Episiotomy

Although midline episiotomy is a strong independent risk factor for third-degree or fourth-degree lacerations,[5] the data are less clear for mediolateral episiotomy. Comparing restrictive episiotomy practices to routine performance, a meta-analysis of eight randomized trials (5,541 women) found that restrictive practices (28% episiotomy rate) were associated with a lower risk of severe perineal trauma (RR, 0.67; 95% CI, 0.49–0.91), posterior perineal trauma (RR, 0.88; 95% CI, 0.84–0.92), need for suture repair of perineal trauma (RR, 0.71; 95% CI, 0.61–0.81), and healing complications at 7 days (RR, 0.69; 95% CI, 0.56–0.85) compared with patients in the routine episiotomy study arm (75% episiotomy rate).[7] Anal sphincter injuries may be reduced with mediolateral episiotomy. The Collaborative Perinatal Project described the association of episiotomy type and OASIS in 24,114 women. Adjusted odds ratios for OASIS increased with midline episiotomy among primiparous women (adjusted OR, 4.2; 95% CI, 1.8–10.0) and multiparous women (adjusted OR, 12.8; 95% CI, 5.4–30.3), whereas mediolateral episiotomy was associated with a reduced risk of OASIS in primiparous women (adjusted OR, 0.4; 95% CI, 0.2–0.9) but had no effect on OASIS in multiparous women.[8] There is only one published RCT comparing rates of OASIS between midline and mediolateral episiotomy in nulliparas.[9] In that study, 12% of women who had a midline episiotomy sustained an OASIS, versus 2% of those who had a mediolateral. In a prospective cohort study of 1302 women who delivered vaginally, and who all received an episiotomy, 426 received midline and 876 mediolateral episiotomy, according to the practitioner’s preferences.[10] Deep perineal tears (which included but were not limited to OASIS) were present in 14.8% of those who had a midline episiotomy versus 7% of those who had a mediolateral episiotomy.

Postpartum Management:

Occult OASIS, or laceration of the anal sphincter complex with no clinical findings but later identified by endoanal ultrasonography, has been reported to occur in 27% of women after their first vaginal delivery. Therefore, a high index of suspicion is required and a thorough examination is recommended after delivery.[11]

Anal Mucosal Repair

Expert opinion varies on the technique and suture used to reapproximate the anal mucosa. Interrupted and running suturing techniques have been described. The use of 4-0 or 3-0 Vicryl and chromic have been suggested. There are no comparative studies regarding suture technique or material.[12-13]

Repair of Anal Sphincter

The two accepted techniques in repairing the anal sphincter is end to end and overlapping. A meta-analysis of six randomized controlled studies (588 women) of variable quality that compared end-to-end repair versus overlap repair for a grade 3c or greater laceration found no differences between the two techniques at 12 months in incidence of perineal pain, dyspareunia, or flatal incontinence. However, a lower incidence of fecal urgency (RR, 0.12; 95% CI, 0.02–0.86; one trial, 52 women) and lower anal incontinence scores (standardized mean difference, −0.70; 95% CI, −1.26 to −0.14; one trial, 52 women) were observed in women undergoing overlap repair. The overlap technique was associated with a lower risk of anal incontinence symptoms over 12 months (RR, 0.26; 95% CI, 0.09–0.79; one trial, 41 women). There were no significant difference in quality of life or in anal incontinence symptoms (either flatal incontinence or fecal incontinence) 36 months after repair.[14] The only randomized controlled trial that compared suture types in the repair of anal sphincter injury (with no attempt made to separately identify the internal anal sphincter) did not find a difference between 3-0 Vicryl or 3-0 PDS suture-related morbidity at 6 weeks postpartum or level of bowel continence or quality-of-life score at 3 months postpartum.[15] Expert opinions states that 2-0 or 3-0 Vicryl or PDS is acceptable to repair the anal sphincter.

Postoperative Antibiotic Use

In a randomized controlled trial in which patients received a single dose of a second-generation cephalosporin (cefotetan or cefoxitin) versus placebo at the time of repair for OASIS, there were significantly lower rates of postpartum wound complications at 2 weeks with antibiotic use compared with placebo (8% versus 24%, *P*=.04).[16] In two cohort investigations from the same institution, intrapartum antibiotics were protective in a retrospective (OR, 0.29; 95% CI, 0.14–0.59) and prospective study (adjusted OR, 0.50; 95% CI, 0.27–0.94).[17-18]

Constipation Avoidance

Management of post-OASIS constipation was studied in one randomized controlled trial that compared 3 days of an oral laxative (lactulose) versus a constipating regimen (codeine phosphate). The use of an oral laxative was associated with significantly less pain (median pain visual analog scale 2 versus 3 at the time of first bowel movement, *P*<.01) and earlier bowel movements (median 2 days versus 4 days, *P*<.01), compared with the constipating regimen. Stool softeners and oral laxatives should be prescribed to women who sustain OASIS and counseling postpartum should include discussing ways to avoid constipation.[19] Expert opinion suggests the use of mineral oil and a low residue diet to aid in passage of stools and reduce the risk of repair breakdown.[20]

Pain control

A meta-analysis showed no improvement in pain control when topical anesthetics were compared with placebo.[21] There is limited evidence to support the effectiveness of local cooling treatments, including ice packs, cold gel pads, and cold or iced baths, applied to the perineum after childbirth to relieve pain. The largest trial available noted that the group that received ice packs reported significantly less moderate or severe pain between 24 hours and 72 hours after birth compared with women who received no treatment (RR, 0.61; 95% CI, 0.41–0.91, 208 women).[22] Rectal suppositories (diclofenac and indomethacin) when compared with placebo in the first 24 hours after birth did not influence patients' numerical pain score; however, the use of additional analgesia for perineal pain was reduced (RR, 0.31; 95% CI, 0.17–0.54, one trial, 89 women).[23] Rectal suppositories should be used with caution as to not disrupt the repair. Nonsteroidal anti-inflammatory or opiate agents can be offered for pain control, but should be coupled with oral laxatives and stool softeners to help mediate the significant constipating adverse effects of these medications.

Urinary retention

Women who sustain severe perineal trauma should be monitored for urinary retention. In a recent study of postpartum urinary retention, 33% of women were found to have an OASIS injury. Spontaneous voiding should be carefully monitored, and women that are unable to pass urine or develop discomfort due to bladder distention require prompt evaluation.[24-25]

Hospital length of stay

There is currently no data determining the appropriate length of hospital stay for patients who undergo an OASIS. Expert opinion suggests that the patient is evaluated daily while admitted.

Postpartum Follow Up

Although there are no standard guidelines for follow up, because of the increased rate of wound complications in the short-term postpartum period, expert opinion recommends early and consistent follow-up to reduce the rate of hospital readmissions.[18]

Pelvic Floor Muscle Exercises

Pelvic floor exercises performed with a vaginal device that provides resistance or feedback may decrease postpartum urinary incontinence, but the effect on anal incontinence has been mixed with no demonstration of a durable long-term effect.[26]

Summary of Recommendations

Intrapartum Recommendations:

1. Restricted use of episiotomy is recommended (Level A)

2. Can consider applying warm compress to the perineum during the 2nd stage of labor to reduce the risk of perineal trauma. (Level A)
3. Perineal massage may also reduce 3rd and 4th degree lacerations and can be considered. (Level B)
4. If an episiotomy is indicated, can consider a mediolateral approach rather an midline (Level B)

OASIS Repair Recommendations

1. A single dose of a 2nd generation cephalosporin such as cefotetan or cefoxitin should be given at the time of repair (Level A).
2. A thorough vaginal and perineal examination is recommended and those with a tear should have a rectal examination for OASIS prior to repair (Level B).
3. When there is a full thickness anal sphincter injury, it should be repaired in either end to end or overlapping fashion with 2-0 or 3-0 PDS or Vicryl. For grade 3a or partial thickness grade 3b injuries, end to end reapproximation should be performed (Level A).
4. The anal mucosa should be closed in either a running or interrupted fashion with 4-0 or 3-0 Vicryl or Chromic suture (Level C).

Postoperative Management Recommendation

1. Patients should be screened for preexisting constipation or risk factors (Level C).
2. Oral mineral oil use (1-2 tablespoons daily) as well as a low residue diet may facilitate bowel movement consistency and ease of passage (Level C).
3. Oral laxatives and/or stool softeners should be used to prevent constipation. Bulking agents should not be used concomitantly (Level A).
4. NSAIDs and Tylenol should be mainstay of pain management and narcotics should be limited due to constipating side effects. If narcotics are used, they should be coupled with oral laxatives (Level B).
5. Ice pack should be used for adjuvant pain control (Level B).
6. Close monitoring for urinary retention is recommended (Level C).
7. Length of hospital stay should be determined based on current hospital protocol and the patient's medical comorbidities. There is no evidence for an increased length of stay due to the presence of an OASIS (Level C).
8. Early and consistent postpartum follow up is recommended (1-2 weeks) (Level C).
9. Pelvic floor physical therapy may be discussed with the patient (Level C).

References:

ACOG Practice Bulletin 198 “Prevention and Management of Obstetric Lacerations at Delivery”. Published on August 22, 2018

1. Jha S, Parker V. Risk factors for recurrent obstetric anal sphincter injury (rOASI): a systematic review and meta-analysis. Int Urogynecol J 2016; 27:849. (Meta-Analysis)
2. Evers EC, Blomquist JL, McDermott KC, Handa VL. Obstetrical anal sphincter laceration and anal incontinence 5–10 years after childbirth. Am J Obstet Gynecol 2012;207:425.e1–6. (Level II-3)
3. Fenner DE, Genberg B, Brahma P, Marek L, DeLancey JO. Fecal and urinary incontinence after vaginal delivery with anal sphincter disruption in an obstetrics unit in the United States. Am J Obstet Gynecol 2003;189:1543–9; discussion 1549–50. (Level II-3)
4. Friedman AM, Ananth CV, Prendergast E, D’Alton ME, Wright JD. Evaluation of third-degree and fourth-degree laceration rates as quality indicators. Obstet Gynecol 2015;125:927–37. (Level II-3)
5. Pergialiotis V, Vlachos D, Protopapas A, Pappa K, Vlachos G. Risk factors for severe perineal lacerations during childbirth. Int J Gynaecol Obstet 2014;125:6–14. (Meta-Analysis)
6. Aasheim V, Nilsen AB, Lukasse M, Reinar LM. Perineal techniques during the second stage of labour for reducing perineal trauma. Cochrane Database of Systematic Reviews 2011, Issue 12. Art. No.: CD006672. DOI: 10.1002/14651858.CD006672.pub2. (Meta-Analysis)
7. Carroli G, Mignini L. Episiotomy for vaginal birth. Cochrane Database of Systematic Reviews 2009, Issue 1. Art. No.: CD000081. DOI: 10.1002/14651858.CD000081.pub2. (Meta-Analysis)
8. Shiono P, Klebanoff MA, Carey JC. Midline episiotomies: more harm than good? Obstet Gynecol 1990;75:765–70. (Level II-3)
9. Coats PM, Chan KK, Wilkins M, Beard RJ. A comparison between midline and mediolateral episiotomies. Br J Obstet Gynaecol 1980;87:408–12. (Level I)
10. Sooklim R, Thinkhamrop J, Lumbiganon P, Prasertcharoensuk W, Pattamadilok J, Seekorn K, et al. The outcomes of midline versus medio-lateral episiotomy. Reprod Health 2007;4:10. (Level II)
11. Oberwalder M, Connor J, Wexner SD. Meta-analysis to determine the incidence of obstetric anal sphincter damage. Br J Surg 2003;90:1333–7. (Meta-Analysis)
12. Sultan AH, Thakar R. Lower genital tract and anal sphincter trauma. Best Pract Res Clin Obstet Gynaecol 2002;16:99–115. (Level III)
13. Gilstrap LC 3rd, Cunningham FG, Vandorsten JP. Operative obstetrics. 2nd ed. New York (NY): McGraw-Hill; 2002. (Level III)
14. Fernando RJ, Sultan AH, Kettle C, Thakar R. Methods of repair for obstetric anal sphincter injury. Cochrane Database of Systematic Reviews 2013, Issue 12. Art. No.: CD002866. DOI: 10.1002/14651858.CD002866.pub3. (Meta-Analysis)
15. Williams A, Adams EJ, Tincello DG, Alfirevic Z, Walkinshaw SA, Richmond DH. How to repair an anal sphincter injury after vaginal delivery: results of a randomised controlled trial. BJOG 2006;113:201–7. (Level I)

16. Duggal N, Mercado C, Daniels K, Bujor A, Caughey AB, El-Sayed YY. Antibiotic prophylaxis for prevention of postpartum perineal wound complications: a randomized controlled trial. Obstet Gynecol 2008;111:1268–73. (Level I)
17. Stock L, Basham E, Gossett DR, Lewicky-Gaupp C. Factors associated with wound complications in women with obstetric anal sphincter injuries (OASIS). Am J Obstet Gynecol 2013;208:327.e1–6. (Level II-3)
18. Lewicky-Gaupp C, Leader-Cramer A, Johnson LL, Kenton K, Gossett DR. Wound complications after obstetric anal sphincter injuries. Obstet Gynecol 2015;125:1088–93. (Level II-2)
19. Mahony R, Behan M, O'Herlihy C, O'Connell PR. Randomized, clinical trial of bowel confinement vs. laxative use after primary repair of a third-degree obstetric anal sphincter tear. Dis Colon Rectum 2004;47:12–7. (Level I)
20. Delancey JOL, Berger MB. Surgical approaches to postobstetrical perineal body defects (rectovaginal fistula and chronic third and fourth-degree lacerations). Clin Obstet Gynecol. 2010;53(1):134–144. (Level III)
21. Hedayati H, Parsons J, Crowther CA. Topically applied anaesthetics for treating perineal pain after childbirth. Cochrane Database of Systematic Reviews 2005, Issue 2. Art. No.: CD004223. DOI: 10.1002/14651858.CD004223.pub2. (Meta-Analysis)
22. East CE, Begg L, Henshall NE, Marchant PR, Wallace K. Local cooling for relieving pain from perineal trauma sustained during childbirth. Cochrane Database of Systematic Reviews 2012, Issue 5. Art. No.: CD006304. DOI: 10.1002/14651858.CD006304.pub3. (Meta-Analysis)
23. Hedayati H, Parsons J, Crowther CA. Rectal analgesia for pain from perineal trauma following childbirth. Cochrane Database of Systematic Reviews 2003, Issue 3. Art. No.: CD003931. DOI: 10.1002/14651858.CD003931. (Meta-Analysis)
24. Ching-Chung L, Shuenn-Dhy C, Ling-Hong T, Ching-Chang H, Chao-Lun C, Po-Jen C. Postpartum urinary retention: assessment of contributing factors and long-term clinical impact. Aust N Z J Obstet Gynaecol 2002;42:365–8. (Level II-2)
25. Glavind, K. and Bjork, J. Incidence and treatment of urinary retention postpartum. *Int Urogynecol J*. 2003; 14: 119–121 (Level II-3)
26. Harvey MA. Pelvic floor exercises during and after pregnancy: a systematic review of their role in preventing pelvic floor dysfunction. J Obstet Gynaecol Can 2003;25:487–98. (Meta-Analysis)
