# Supplementary material for: Obstetric Anal Sphincter Injury Care Bundle: A Quality Improvement Initiative
Source: Int Urogynecol J. 2024 Sep 27;35(11):2125–30. doi: 10.1007/s00192-024-05885-2 (PMC11638415; doi:10.1007/s00192-024-05885-2)
Supplement: Supplementary file 2 — Supplementary file2 (PDF 646 KB) [file 192_2024_5885_MOESM2_ESM.pdf]

Electronic Medical Record Order Set (ordered at time of delivery)

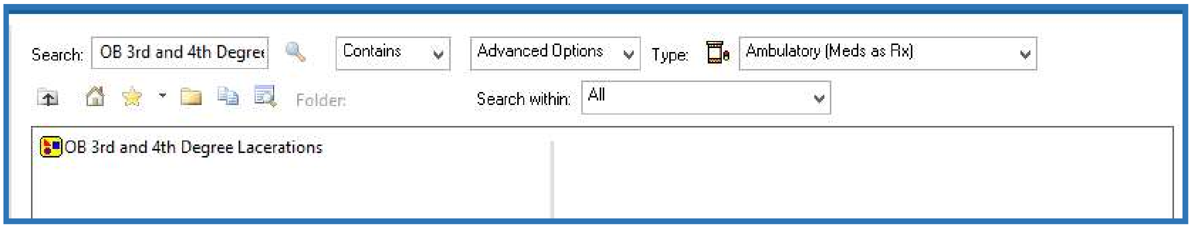

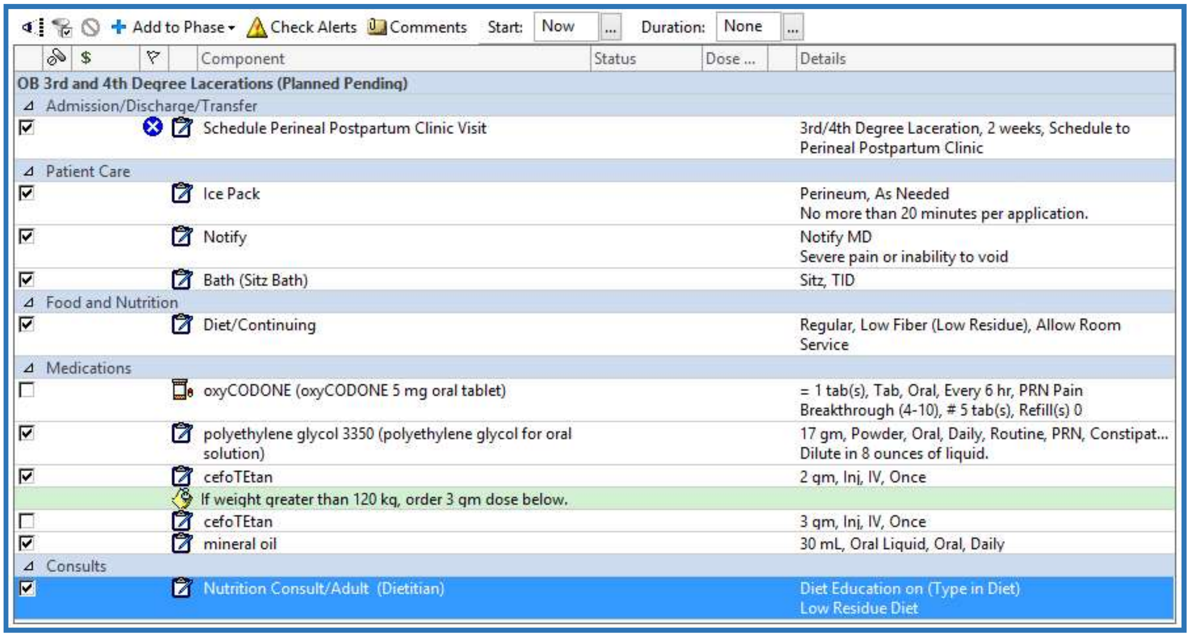

Key Order Set Components

- 2gm Cefotetan IV once given at the time of repair
- Schedule Postpartum Perineal Clinic Visit 2 weeks after delivery
- Ice pack to perineum
- Notify physician of severe pain or inability to void
- Sitz bath treatment three times daily for perineal irrigation
- Low Residue Diet
- Nutrition consult for low residue diet education
- 30mL mineral oil by mouth daily
- 17gm polyethylene glycol diluted in 8oz water by mouth daily

Patient Discharge Education

Care of a Perineal Tear

A perineal tear is a tear in the tissue between the opening of the vagina and the anus (perineum). A perineal tear, also called a laceration, usually happens during vaginal birth. This can happen as the baby emerges from the birth canal or if a surgical cut (episiotomy) is made. The type of perineal tear is based on the depth and length of the laceration. The four types are:

- First degree. This is a shallow tear at the edge of the vaginal opening that goes slightly into the perineal skin.
- Second degree. Tearing in the second degree is a deeper tear of the vaginal opening and perineal tissues. It may also include the tearing of a muscle just under the perineal skin.
- Third degree. Tearing in the third degree extends through the perineal skin and tissues and continues into the muscle of the anus (anal sphincter).
- Fourth degree. Tearing in the fourth degree extends through the perineal tissues, muscle, anus, and into the rectum.

If needed, perineal tears are sutured immediately after the baby's birth. Some perineal tears may not be stitched (sutured) closed depending on their location and appearance.

What are the risks?

Depending on the type of perineal tear you have, you may be at risk for:

- Developing a collection of blood in the perineal tear area (hematoma).
- Pain when you urinate or have a bowel movement.
- Infection at the site of the tear.
- Trouble controlling urination or bowel movements (incontinence).
- Painful sex.

How to care for a perineal tear

Wound care

- Follow instructions from your health care provider about how to take care of your tear.
- Leave the sutures in place as told by your health care provider. Sutures used to repair your tear are absorbable and do not need to be removed.
- Wear a sanitary pad as told by your health care provider.
- Check your wound every day for signs of infection. Check for:
  - Redness, swelling, or pain.
  - Warmth.
  - Pus or a bad smell.
- Take sitz baths as told by your health care provider to speed up healing. In a sitz bath, you sit down in warm water.

Managing pain

- If directed, put ice on the affected area. To do this:
  - Put ice in a plastic bag.
  - Place a towel between your skin and the bag.
  - Leave the ice on for 20 minutes, 2–3 times a day.
  - Remove the ice if your skin turns bright red. This is very important. If you cannot feel pain, heat, or cold, you have a greater risk of damage to the area.
- Apply a numbing spray to the perineal tear as told by your health care provider. This may help with discomfort.
- Take or apply over-the-counter and prescription medicines only as told by your health care provider.

- If told by your health care provider, use witch hazel-containing hemorrhoid treatment pads on top of your sanitary pad. These help with swelling and discomfort.
- If it is comfortable, sit on an inflatable ring or pillow.

General instructions

- Instead of wiping, use a squirt bottle to squeeze warm water on your perineum after urinating to clean the area. Do this from front to back. Pat the area gently to dry it.
- **Do not** have sex, use tampons, or place anything in your vagina for at least 6 weeks or as told by your health care provider.
- Keep all follow-up visits. Your health care provider will check how the tear is healing.

Contact a health care provider if:

- Medicine does not help your pain.
- It hurts to urinate.
- You have redness, swelling, or pain around your tear.
- Your tear feels warm to the touch.
- You have pus or a bad smell coming from your tear.
- You have a fever.
- You have more bleeding.

Get help right away if:

- Your tear opens.
- You cannot urinate.
- You have severe pain.

Summary

- A perineal tear is a tear in the tissue between the opening of the vagina and the anus (perineum).
- Follow instructions from your health care provider about how to take care of your tear.
- Keep all follow-up visits. Your health care provider will check how the tear is healing.
- Get help right away if your tear opens, you cannot urinate, or you have severe pain.

This information is not intended to replace advice given to you by your health care provider. Make sure you discuss any questions you have with your health care provider.

Low-Residue Diet

This diet is designed to reduce the frequency and volume of fecal output to allow for healing of your perineum.

Guidelines:

- Continue taking daily mineral oil (30mL) and polyethylene glycol (17g in 8oz water) until your follow up clinic appointment
- Buy breads and cereals made from refined wheat and rice. Avoid whole-grain products with added bran.
- Remove skin from vegetables and fruits before cooking.
- Avoid any food made with seeds, nuts, and raw or dried fruits.
- Limit milk and milk products to 2 cups daily.

Sample Menu:

Breakfast:

cranberry juice (1/2 C) puffed rice cereal (3/4 C) canned peaches (1/2 C) white toast (2 slices)

margarine (2 tsp) jelly (1 tbsp) 2% milk (1/2 C)

Lunch:

lean beef patty (3 oz) hamburger bun without seeds mustard (1 tbsp) ketcup (1 tbsp) canned fruit cocktail (1/2 C) vanilla wafer cookies (2) 2% milk (1 C)

Dinner:

strained tomato juice (1/2 C) breaded baked chicken (3 oz) white rice (1/2 C) cooked carrots (1/2 C) white dinner roll margarine (2 tsp) sherbert (1/2 C) 2% milk (1 C)

Afternoon Snack:

applesauce (1/2 C) saltine crackers (2 squares)

Evening Snack:

fruit ice (1/2 C)

FOODS RECOMMENDED:

Breads/Grains:

- Refined breads, toast, rolls, biscuits, muffins, crackers, pancakes, and waffles.
- Enriched white or light rye bread or rolls.
- Saltines, Melba toast
- Refined ready-to-eat cereals such as puffed rice and puffed wheat
- Cooked refined wheat, corn, or rice cereal
- Strained oatmeal, grits and farina
- Refined cold cereals made from rice, corn or oats (Rice Krispies, Cornflakes, Cheerios)
- White rice, refined pasta, macaroni, noodles

Vegetables: Most tender cooked and canned vegetables without seeds such as carrots, asparagus tips, beets, green or waxed beans, pumpkin, spinach, squash (acorn) without seeds, potato (no skin), pureed or cooked strained lima beans, and peas (no skin)

Fruits: Most canned or cooked fruits, fruit cocktail, avocado, canned applesauce, apricots, peaches, pears (all without skin and seeds), pureed plums and ripe bananas Strained fruit juice

Milk/Dairy:

- Milk, mild cheese, cottage cheese
- Yogurt (no berries)
- *limit milk/milk products to 2 cups per day

Meat:

- Ground or well-cooked, tender beef, lamb, ham, veal, pork, fish, shellfish, and organ meats
- Eggs
- Smooth peanut butter

Fat/Snacks:

Margarine, butter, vegetable oils, mayo, cream substitutes, crisp bacon, plain gravies, and salad dressings Broth, strained cream soups (no corn) made with allowed ingredients

Misc:

Salt, soy sauce, ketchup Mild spices in moderation, white sauce Sugar, honey, jelly, syrup Lemon juice, vinegar, vanilla and other flavoring extracts Decaffeinated coffee, herb tea, caffeine-free carbonated beverages and fruit drink

FOODS TO AVOID:

Breads/Grains: Any bread product made with whole-grain flour or graham flour, bran, seeds, nuts, coconut, or raw or dried fruit, cornbread, and graham crackers Any whole-grain, bran, or granola ceral, oatmeal and cereal with seeds, nuts, coconut or dried fruit Bran, barley, brown and wild rice

Vegetables: Raw vegetables and vegetables with seeds, sauerkraut, winter squash, and peas

Fruits: Raw or dried fruit, all berries Prune juice

Milk/Dairy: Yogurt containing fruit skins or seeds Strongly flavored cheeses

Meat: Tough fibrous meats with gristle, shellfish with tough connective tissue Meats prepared with whole-grain ingredients, seeds, or nuts Dry beans, legumes, peas and lentils Chunky peanut butter Raw clams and oysters

Fats/Snacks: Any made with whole-grain flour, bran, seeds, nuts, coconut, or dried fruit Nuts, seeds, and popcorn Pepper, chili pepper and other hot sauces Chocolate, raisins, seeds, seed spices, pickles, olives, nuts, mustards, spicy mustards and ketchups, relish, horseradish, vinegar Highly spiced salad dressings Jam or marmalade with nuts and seeds

Misc: Beverages containing caffeine which is a stomach irritant.
